# Wavelet-Based Visible and Infrared Image Fusion: A Comparative Study

**DOI:** 10.3390/s16060861

**Published:** 2016-06-10

**Authors:** Angel D. Sappa, Juan A. Carvajal, Cristhian A. Aguilera, Miguel Oliveira, Dennis Romero, Boris X. Vintimilla

**Affiliations:** 1 Facultad de Ingeniería en Electricidad y Computación, CIDIS, Escuela Superior Politécnica del Litoral, ESPOL, Campus Gustavo Galindo, Km 30.5 vía Perimetral, Guayaquil 09-01-5863, Ecuador; jcarvajal91@gmail.com (J.A.C.); dgromero@espol.edu.ec (D.R.); boris.vintimilla@espol.edu.ec (B.X.V.); 2 Computer Vision Center, Edifici O, Campus UAB, Bellaterra 08193, Barcelona, Spain; caguilera@cvc.uab.es; 3 Computer Science Department, Universitat Autònoma de Barcelona, Campus UAB, Bellaterra 08193, Barcelona, Spain; 4 Institute for Systems and Computer Engineering, Technology and Science, R. Dr. Roberto Frias 465, Porto 4200, Portugal; m.riem.oliveira@gmail.com; 5 Institute of Electronics and Informatics Engineering of Aveiro, Campus Universitário de Santiago 3810-193 Aveiro, Portugal

**Keywords:** image fusion, fusion evaluation metrics, visible and infrared imaging, discrete wavelet transform

## Abstract

This paper evaluates different wavelet-based cross-spectral image fusion strategies adopted to merge visible and infrared images. The objective is to find the best setup independently of the evaluation metric used to measure the performance. Quantitative performance results are obtained with state of the art approaches together with adaptations proposed in the current work. The options evaluated in the current work result from the combination of different setups in the wavelet image decomposition stage together with different fusion strategies for the final merging stage that generates the resulting representation. Most of the approaches evaluate results according to the application for which they are intended for. Sometimes a human observer is selected to judge the quality of the obtained results. In the current work, quantitative values are considered in order to find correlations between setups and performance of obtained results; these correlations can be used to define a criteria for selecting the best fusion strategy for a given pair of cross-spectral images. The whole procedure is evaluated with a large set of correctly registered visible and infrared image pairs, including both Near InfraRed (NIR) and Long Wave InfraRed (LWIR).

## 1. Introduction

Image fusion is the process of combining information from two or more images of a given scene into a single representation. This process is intended for encoding information from source images into a single and more informative one, which could be suitable for further processing or visual perception. There are two different cases where image fusion takes place: firstly, the case of images obtained from different sensors (multisensory), which could also work at different spectral band (multispectral) (e.g., [1,2]). Secondly, the case of images of the same scene but acquired at different times (multitemporal) (e.g., [3,4]). The current work is focussed on the first case, more specifically, fusing pair of images from the visible and infrared spectra obtained at the same time by different sensors. The evaluations are performed using pairs of images from both visible and near infrared (NIR) and visible and long wave infrared (LWIR) spectra. It is assumed that the images to be fused are correctly registered; otherwise, a process of cross-spectral feature detection and description should be followed in order to find the correspondences between the images (e.g., [5,6,7]).

The usage of cross-spectral imaging has been increasing due to the drop in price of cameras working at different spectral bands. That increase is motivated by the possibility of developing robust solutions that cannot be obtained with single spectral band sensors. These robust solutions can be found in domains such as: driving assistance (e.g., [8]), video surveillance (e.g., [9,10]), face detection (e.g., [11]), thermal inspection (e.g., [12,13]), just to mention a few. The information provided by the cameras working at different spectral bands needs to be fused in order to have a single and compact representation for further processing. For instance, the classical monocular visual odometry problem faced in driving assistance can be robustly tackled by using the result from the fusion of visible an infrared spectrum pair of images. The usage of fused images allows to compute visual odometry even in poor-lighting scenarios, which is an advantage in front of classical approaches that are based on a singe spectral band (e.g., [8,14]). The simultaneous use of images from different spectra can be helpful to improve the performance in the fields mentioned above—video surveillance, face detection and thermal inspection.

During the last decades the image fusion problem has been largely studied, mainly for remote sensing applications (e.g., [2,15,16]). Most of these methods have been proposed to produce a high-resolution multispectral representation from a low-resolution multispectral image fused with a high-resolution panchromatic one. The difference in image resolution is generally tackled by means of multi-scale image decomposition schemes that preserve spectral characteristics but represented at a high spatial resolution. Among the different proposals, wavelet-based approaches have shown one of the best performance by producing better results than standard methods such as the intensity-hue-saturation (IHS) transform technique or principal component analysis (PCA) [17]. Wavelet-based image fusion consists of two stages. Firstly, the given images are decomposed into two components (more details are given in Section 2); secondly, the components from the given images are fused in order to generate the final representation. Hence, the main challenge with wavelet-based fusion schemes lies on finding the best setup for both, the image decomposition approach (*i.e.*, number of levels, wavelet family and its configurations) and the fusion strategy to merge the information from decomposed images into a single representation (e.g., min, max, mean, rand, etc., from the two approximations and details obtained from the given images at elementwise by taking respectively the minimum, the maximum, the mean value, or a random element). The selection of the right setup for fusing the given images will depend on the way the performance is evaluated. Hence, a special care should be paid to the quantitative metric used to evaluate the obtained result, avoiding psychophysical experiments that will result in qualitative values [18].

Different approaches have been proposed in the literature to evaluate fusion results; they can be classified into two categories depending on the existence or not of a reference image [19]. In the case a reference image is available, it can be used as a ground truth to evaluate results by means of quality metrics such as: Root Mean Square Error (RMSE), Peak Signal to Noise Ratio (PSNR), Structural Similarity Index Measure (SSIM), Mutual Information (MI) among others (e.g., [20,21]). Otherwise, when there is no reference image, the quality of the results is indirectly measured through some metrics such as: Entropy (a high entropy value indicates the fused image as rich in information content), Standard Deviation (high values indicate high contrast) and Mutual Information (the larger the value the better quality fused images) (e.g., [22,23]).

This paper addresses the problem of finding the best setup by means of an empirical approach where a large number of configurations (setups) are quantitatively evaluated. The goal is to gain experience that may contribute to support informed decisions of which should be the best setup given a description of the problem. The different configurations are evaluated by means of the usage of four different metrics. Since reference images are not available, quality metrics are adaptations of the ones mentioned above. More specifically: Fused Peak Signal to Noise Ratio (FPSNR), Fused Mutual Information (FMI), Fused Structural Similarity (FSS) and Fused S-CIELAB (FS-CIELAB). Their definitions are presented in Section 3. Essentially, they try to measure how much of the information contained in the given images is also present in the fused one; ideally the fused image should contain all the information from both images. It should be highlighted, as mentioned above, that the current work is focussed on the usage of visible and infrared spectrum (including both NIR and LWIR) images. The usage of these two spectral bands is motivated by the hardware that is already present in different platforms (there are: vehicles with visible and LWIR cameras; videosurveillance platforms that integrate both kind of cameras and thermal inspection devices that capture both visible and thermal information).

The remainder of the paper is organized as follows. Section 2 briefly presents the wavelet-based image fusion framework together with a description of the different configurations evaluated in this study. Section 3 introduces the metrics used to evaluate results; evaluations with pairs of both visible and NIR images and visible and LWIR images are presented in Section 4. Finally, conclusions are given in Section 5.

## 2. Wavelet-Based Image Fusion

**Figure 1:**
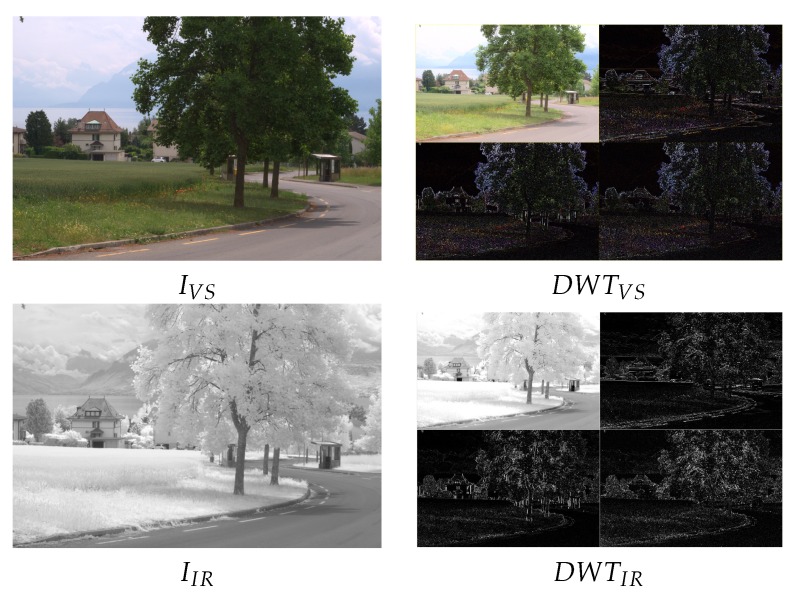
(**Left**) Pair of images (VS-IR) to be fused; (**Right**) DWT decompositions (one level) of the input images.

Wavelet theory has been largely studied in digital signal processing and applied to several subjects (from noise reduction [24] to texture classification [25], just to mention a couple). At this section, the basic concepts and elements of Discrete Wavelet Transform (DWT) in the context of image fusion are introduced. Let $I_{VS}$ and $I_{IR}$ be the original images, of m×n pixels, in the visible (VS) and infrared (IR) spectra respectively (IR refers to both NIR and LWIR images). Let $I_{F}$ be the image, also of m×n pixels, resulting from their fusion. In the wavelet-based image fusion, the given images are decomposed at their corresponding approximation (*A*) and detail (*D*) components, which correspond to the lowpass and highpass filtering for each decomposition level. These decompositions can be represented through sub-images. The detail representations correspond to the vertical details (VD), horizontal details (HD) and diagonal details (DD) respectively. Figure 1 (Right) depicts illustrations of one level DWT decompositions obtained from the original images Figure 1 (Left) (different approaches used to decompose the given images are introduced in Section 2.1).

**Figure 2:**
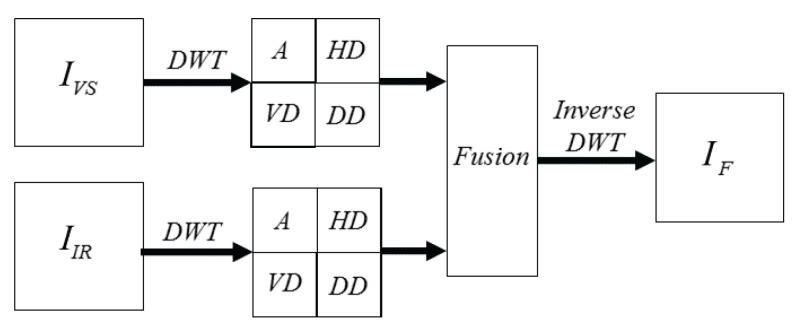
Illustration of DWT based fusion scheme.

Once the coefficients (approximations and details) from each decomposition level are obtained a fusion scheme is applied to *catch* the most representative information from each representation. The most widely used fusion schemes proposed in the literature to merge the information are reviewed in Section 2.2. Finally, the inverse DWT is applied to the result in order to obtain the sought fused image ($I_{F}$). Figure 2 presents a classical DWT based image fusion pipeline.

### 2.1. Discrete Wavelet Transform (DWT)

At this section basic concepts of discrete wavelet transform are introduced. The DWT can be represented as a bank of filters, where at each level of decomposition the given signal is split up into high frequency and low frequency components. The low frequency components can be further decomposed until the desired resolution is reached. If multiple levels of decomposition are applied, it is referred to as multiresolution decomposition. Although there is no rule, in general, in the image fusion problem just one level of decomposition is considered. In the current work, the optimum number for the level of decomposition is found by evaluating different configurations.

In discrete wavelet transform theory several wavelet *families* have been proposed in the literature. Each family has a wavelet function and a scaling function. These two functions act as a high pass filter (the wavelet function) and a low pass filter (the scaling function). A wavelet family is normally represented by only its wavelet function. Both of these functions must satisfy some conditions to ensure that the transform can be done. More details about the conditions that must be satisfied can be found in [26]. Within each of these families we have some subclasses that depend on the number of vanishing moments in the wavelet function. This is just a mathematical property that we can directly relate to the number of coefficients. Each of these wavelet functions and their subclasses represent a different way of decomposing a signal; several of these wavelet functions have been considered in the current cross-spectral image fusion evaluation study.

**Figure 3:**
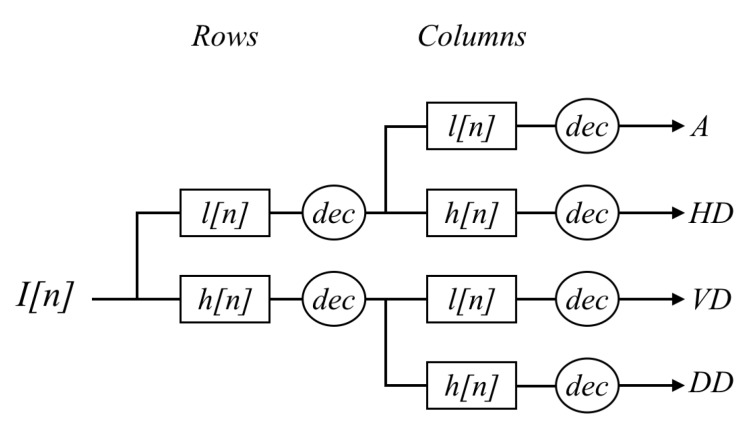
Two dimensional wavelet decomposition scheme (*l*: low pass filter; *h*: high pass filter; *dec*: decimation).

In addition to the wavelet function family, there also exist different variations to the way the approximation and detail coefficients are obtained. These variations are related with the sampling of the signal after the transformation is applied. This result in a larger number of setups to be considered in the current evaluation study. The possible variations are as follows:

**Decimated**: In this case the approximation and details images are downsampled after each level of decomposition (in case of multi level decomposition), keeping one out of every two rows and columns. As previously mentioned, the wavelet and scaling functions can be viewed as high and low pass filters, respectively. Because these filters are one dimensional, when dealing with 2D images the process consists of applying these two filters first to the rows and then to the columns. Figure 3 presents an illustration of this concatenation of high (*h*) and low (*l*) pass filters applied to the rows and columns of a given image in order to obtain the approximation (*A*) and details (*HD, VD, DD*).

This produces the approximation image (low pass filtering to both the rows and columns) and the detail images shown in Figure 1 (right) at half the size of the original input image. When applying the inverse transform, which needs to be done after the fusion stage (see Figure 2), the approximation and details images are first upsampled and then the inverse filter is applied.

The main problem of using decimation in image fusion is that it tends to introduce artifacts when the images to be fused are not correctly registered; the same problem happens for features that do not have horizontal or vertical orientation.

**Undecimated**: In this case instead of downsampling the resulting approximation and detail images, the filters are upsampled. This produces approximation and details images of the same size as the original ones but with half the resolution. In this case, when doing the inverse transform, the filters are downsampled.

This way of applying low and high pass filters solves the problems derived from the shifts in the original images, which were originally caused by the down sampling of the approximation and details, but problems from the features without horizontal or vertical orientation still remain.

**Non separated**: The issue with the horizontal and vertical orientated features is due to the fact that rows and columns are separately filtered. A solution to this problem is to use a two dimensional wavelet filter derived from the scaling function. This will result in an approximation image obtained from the filtering and one detail image that can be obtained from the difference of the original image with the approximation image. The results are similar to the one obtained with the undecimated approach, in the sense that the resolutions will decrease with each level of decomposition because the filter is upsampled.

Since this approach does not imply a down sampling, there is no issue with the shifts between the original images; additionally, since 2D filters are applied, instead of applying 1D filters to the rows and columns respectively, the orientation problem is reduced [27].

### 2.2. Fusion Strategies

Once the given images are split up into the corresponding approximation images and details images (*i.e.*, horizontal details, vertical details and diagonal details) the fused image ($I_{F}$) is obtained by using a merging scheme that takes into account the approximation and detail information from both images—a correct registration is assumed. Some of the most used merging schemes (e.g., [26,28]) are summarized below:

**Substitutive wavelet fusion**: in this scheme the information from one image is completely replaced with information from the other image. In other words, the approximation from one image is merged with the detail of the other image. In the current work the two possible configurations have been considered: ($A_{VS},D_{IR}$) and ($A_{IR},D_{VS}$). Once the information is merged the inverse transform is computed to obtain $I_{F}$.

**Additive wavelet fusion**: as indicated by the name, at this scheme the approximations from one image are added to the other one. The same happens for the detail information. If multiple decompositions were applied, the details at each resolution level are added. Finally, after merging the information the inverse transform is performed resulting in the sough $I_{F}$. In our implementation this scheme is implemented by considering the mean value, instead of just the result from the addition.

**Weighted models**: at this scheme a user tuned merging strategy is applied. Depending on the application and the kind of input images approximations and details are combined according to some statistic values (μ,σ) or according to some other relevant criteria. At the current work, since input images are of the same resolution, and we intend to evaluate the performance of fusion based on DWT of infrared and visible images in a general way, this scheme is not considered.

Other schemes have been proposed in the literature, which somehow can be considered as combinations of the ones presented above; for instance in this work a strategy that considers the minimum value from each image (approximation or detail images), the maximum value or a random selection was also considered.

## 3. Evaluation Metrics

Quantitative evaluation of the quality of fused images has been an active research topic in recent years (e.g., [19,21]). As mentioned above, proposed approaches can be classified into two categories, depending whether a reference image is given or not. In the current work, there is no reference image. Nonetheless, we propose to adapt quality metrics generally used when reference images are provided, instead of using indirect metrics such as entropy or standard deviation based approaches [19]. The proposed adaptations and their descriptions are as follows.

**Fused Peak Signal to Noise Ratio (FPSNR)**: is based on the widely used metric (PSNR), which is computed from the ratio between the number of gray levels (*L*) in the image and the mean squared error between the intensity value of the fused image and the reference one. In our case, since there is no reference image, this value is computed twice, once with the visible image and once with the infrared image used as input information. Then, the average value is considered:

(1) $$\begin{array}{c} {\text{FPSNR}}_{VS-IR}^{F}=\left({\text{PSNR}}_{F,VS}+{\text{PSNR}}_{F,IR}\right)/2 \end{array}$$

where ${\text{PSNR}}_{F,k}=20log_{10}\left(L^{2}/\frac{1}{mn}\sum_{i=1}^{m}\sum_{j=1}^{n}{\left(I_{k}\left(i,j\right)-I_{F}\left(i,j\right)\right)}^{2}\right),k=\left\{VS,IR\right\}$.

**Fused Mutual Information (FMI)**: has been proposed in [29] and later on improved in [22], where a faster approach is proposed—the acronym FMI in the original paper refers to Feature Mutual Information, but here we propose to update it to our notation. This metric evaluates the performance of the fusion algorithm by measuring the amount of information carried from the source images to the fused image by means of mutual information (MI). MI measures the degree of dependency between two variables *A* and *B*, by measuring the distance between the joint distribution $p_{AB}\left(a,b\right)$ and the distribution associated with the case of complete independence $p_{A}a\cdot p_{B}b$, by means of the relative entropy (see [29] for more details):

(2) $$\begin{array}{c} {\text{FMI}}_{VS-IR}^{F}=\left({\text{MI}}_{F,VS}/\left(H_{F}+H_{VS}\right)+{\text{MI}}_{F,IR}/\left(H_{F}+H_{IR}\right)\right)/2 \end{array}$$

where $H_{k}$, with $k=\left\{VS,IR,F\right\}$, are the histogram based entropies of the visible, infrared and fused images respectively as presented in [29].

**Fused Structural Similarity (FSS)**: is based on the work presented in [30]; the structural similarity between $I_{1}$ and $I_{2}$ is defined as $SS_{I_{1},I_{2}}=SSIM\left(I_{1},I_{2}\right)$, where $SSIM\left(I_{1},I_{2}\right)=\frac{1}{N}\sum_{j=1}^{N}SSIM\left(a_{j},b_{j}\right)$ is the Structural SIMilarity (SSIM) index proposed in [21]. Hence, the FSS is computed as:

(3) $$\begin{array}{c} {\text{FSS}}_{VS-IR}^{F}=\left({\text{SS}}_{F,VS}+{\text{SS}}_{F,IR}\right)/2 \end{array}$$

**Fused S-CIELAB (FS-CIELAB)**: is based on the spatial cielab (S-CIELAB) approach presented in [31]. Although this approach has been originally proposed for measuring color reproduction errors in digital images, it has been also used for measuring fusion results in color images [32]. It is computed as:

(4) $$\begin{array}{c} {\text{FS-CIELAB}}_{VS-IR}^{F}=\left({\text{S-CIELAB}}_{F,VS}+{\text{S-CIELAB}}_{F,IR}\right)/2 \end{array}$$

For details about S−CIELAB evaluation metric see [31].

All the previous approaches are direct adaptations where just the average between the resulting quantitative evaluations between each source image and the resulting fused one is computed. More elaborated metrics, computed at a pixel-wise level, could be computed and maybe would result in a more representative value of the algorithm’s performance. Such study is out of the scope of current work and is considered future work.

## 4. Experimental Results

The proposed comparative study has been carried out using the public data set presented in [33], which consists of 477 pair of cross-spectral images (NIR and visible spectra) distributed into 9 categories: Country(52), Field(51), Forest(53), Mountain(55), Old Building(51), Street(50), Urban(58), Water(51)—for more details about the data set see [33]. Additionally, pairs of cross-spectral images (LWIR and visible spectra) from [8], have been considered to evaluate the obtained results. As presented in Section 2 different setups have been tested, both in the DWT decomposition stage as well as in the fusion strategy (see Figure 2). Table 1 presents the variables evaluated in the current study; this includes: wavelet family, decomposition level and fusion scheme (for both approximation and details). The different values for these variables define the setups used for both the DWT decomposition and the Inverse DWT; in all the cases the decimated option has been considered. A more detailed description about the evaluated Wavelet families is provided in Table 2. Regarding the fusion strategy, four different options have been considered: mean (mean value between approximation coefficients and mean value between detail coefficients); max (the coefficients with maximum value is selected, in both cases approximation and details); min (the coefficients with minimum values are selected); rand (coefficients of approximation and details are randomly selected). Trying all the different combinations it results in a family of 2592 possibilities, which were tested using just a pair of images from [33]. The obtained results were later on validated with a larger set of images (we randomly select 2 pairs of images per category from [33] and the comparative study is repeated just with the best 3% of configurations, but not with all the 2592 possibilities). Finally, pairs of cross-spectral images from [8] have been also considered to evaluate the obtained results.

**Figure 4:**
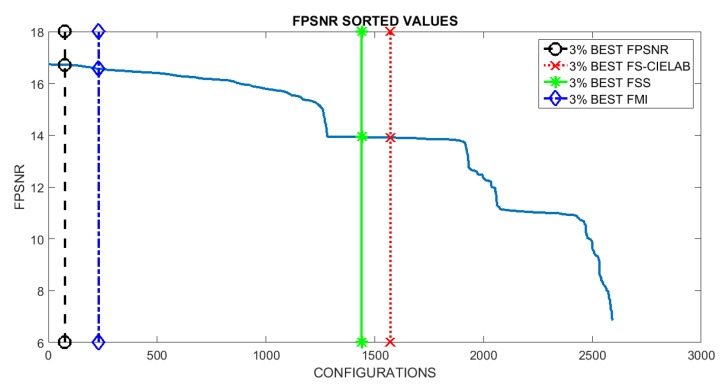

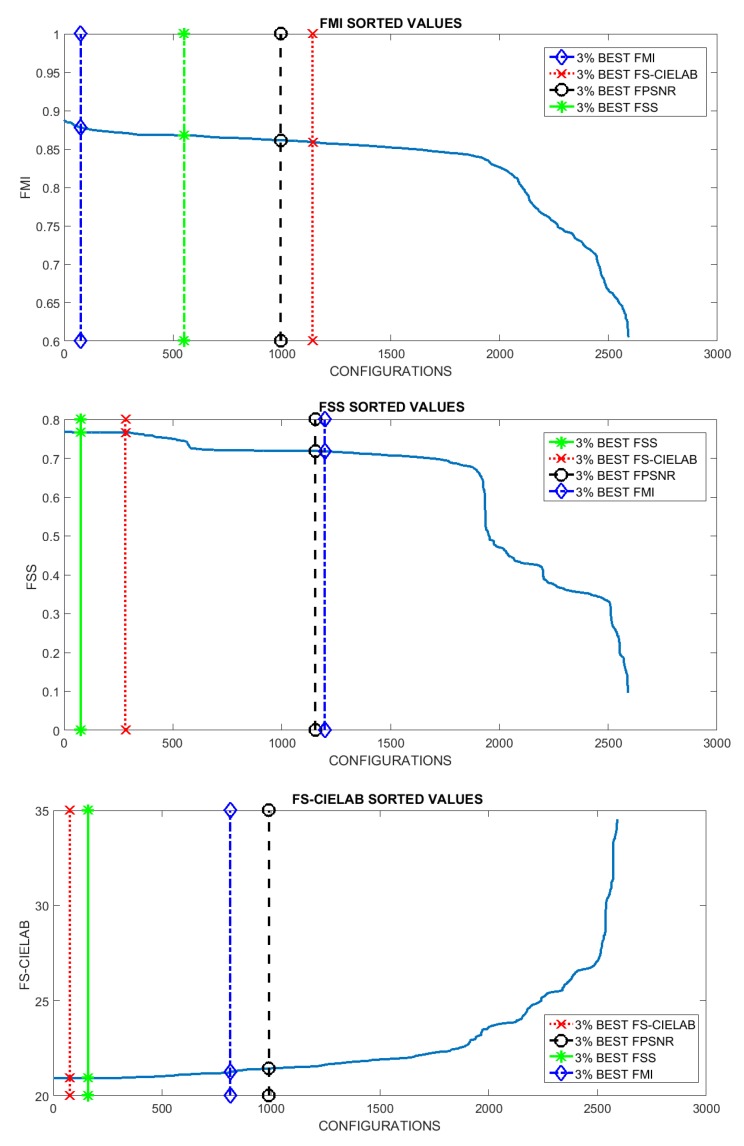
Results sorted according to the metric used for the evaluation (note FS-CIELAB is a dissimilarity measure, meaning that the smaller the score the better the metric quality).

**Figure 5:**
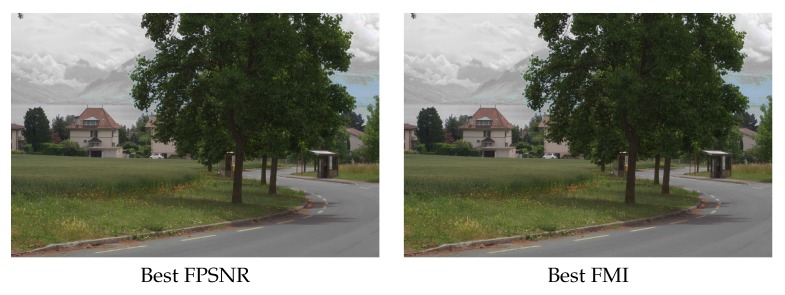

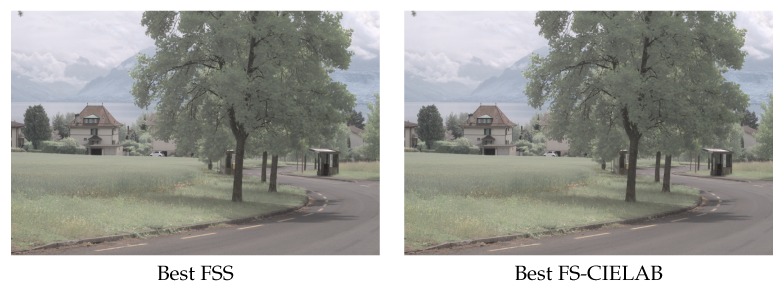
Best DWT fusion results according with the evaluated metrics.

**Figure 6:**
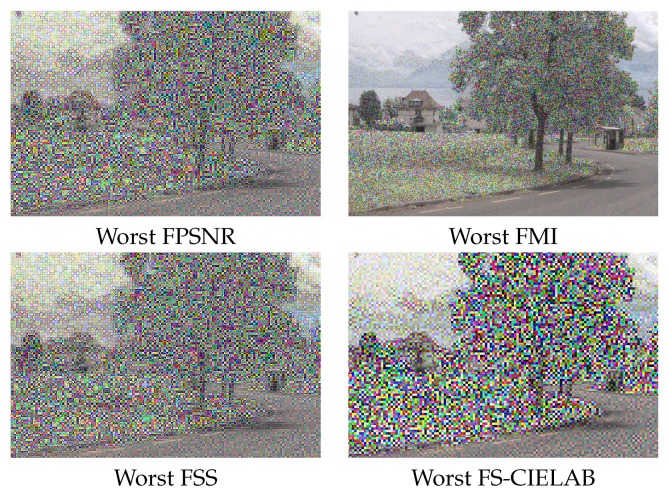
Worst DWT fusion results according with the evaluated metrics.

The VS and IR images are fused by applying the fusion scheme three times, one per each (RGB) channel of the visible image. In other words, the DWT based fusion is applied to the following pairs: ($I_{VS_{R}},I_{IR}$), ($I_{VS_{G}},I_{IR}$) and ($I_{VS_{B}},I_{IR}$). In this way the resulting fused image ($I_{F}$) can be represented in the (RGB) color space. The evaluation consisted of fusing a pair of images (the ones presented in Figure 1) using all the possible setups. This results in 2592 images ($I_{F_{i}}$, where *i* is the index associated with a given setup). These images are then evaluated using the four evaluation metrics presented in Section 3. Results are sorted and plotted in Figure 4. Vertical lines represent the position that includes the setups corresponding to the best 3% at each evaluation metric. This 3% of configurations correspond to the top first 3% when the given evaluation metric is considered; however, when the performance of these setups is evaluated according to other metrics it is not in the top best 3% but it spans covering a larger set of configurations in the sorted list. The (3%) value has been selected just as a reference to study the behavior of best setups; in other words, to analyze how sensitive are the best setups to the used metrics. Just as illustrations Figure 5 and Figure 6 depict the best and worst results according with each of the four evaluation metrics presented in Section 3. These best and worst results correspond to the first and last configurations presented in the four plots in Figure 4.

Looking at the resulting plots we try to identify the evaluation metric that should be used to select the best setups (*i.e.*, configurations). Hypothetically, the best configurations at a given metric should be also among the best ones when they are considered under a different evaluation metric. Although this is not exactly the case if we look at Figure 4, we can appreciate that in the last three plots, the ones corresponding to FMI, FSS and FS-CIELAB metrics, there is not a big change in performance when different top best 3% configurations are considered. This behavior does not hold when the FPSNR metric is considered. In this case we can appreciate that there is a larger decrease in performance when the top best configurations from FSS and FS-CIELAB metrics are considered. Table 3 presents the percentages of decrease when the different metrics are considered. In summary, we can conclude that selecting the configurations, based on FPSNR or FMI metrics, represent a good choice since these configurations return good results even when other metrics are considered.

**Figure 7:**
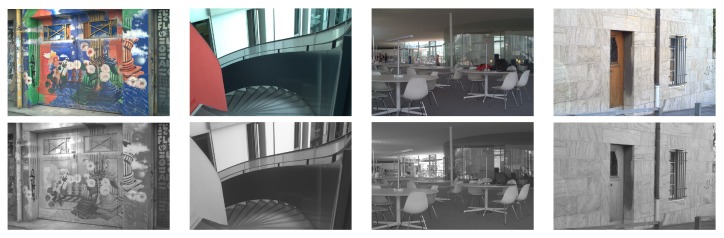
Four pairs of images from the subset used for validation: (**Top**) Visible spectrum images; (**Bottom**) NIR images.

In order to validate the obtained results, a new set of images corresponding to other categories from [33], has been evaluated—2 pairs (VS and NIR) of images per category are randomly selected (some of them are depicted in Figure 7). In this case, the top best setups (configurations) from the FPSNR evaluation metric have been considered since they are the most stable when considered under other evaluation metrics. Results obtained from this new data set have similar behavior than the ones obtained in the pair of test image (see Figure 1); actually, the values have the same order of magnitude. The only exception comes when measuring FS-CIELAB with pair of images from severely different environments than the pair of image used in the study (the test image). The test images were selected from the country folder, and these exceptions come when measuring FS-CIELAB from environments such as old building, street and urban categories. This leads us to the conclusion that FS-CIELAB behaves differently according to the nature of the environment. This conclusion needs to be studied and validated in a further research. In summary, our selected best configurations behave good enough when other set of images are considered; hence, we can conclude they are the best options for fusing cross-spectral images independently of the environment and nature of the images.

Looking at the setups with best results we can observe that: (1) using **one level of decomposition** is enough for the fusion of images; even though in some cases another level may perform similarly or slightly better, the very small difference in the measurement value does not justify the usage of further decomposition levels; (2) the **reverse biorthogonal wavelet family** is the one that appears more times in the set of best configurations, independently of the metric selected for the evaluation. From the reverse biorthogonal family, the rbio5.5 was the best one. Surprisingly, when counting the number of times each family appears in the worst configurations (we did a similar study but with the worst 3% of configurations), the reverse biorthogonal appears in a greater number as well. This behavior can be easily understood in combination with the next point (selection of fusion strategy); (3) regarding the fusion strategy **the approximation weights much more than the details**, as expected, and the selection of approximation strategy varies according to metric selected for evaluating the results. For FS-CIELAB and FSS the mean between both approximation images (NIR and RGB) was always the best selection; for FPSNR it was distributed almost evenly between the minimum and maximum between both approximation images; in other words, independently of the selection (min or max) a good result is obtained. Finally, for FMI, the minimum was always the best choice. The worst configurations, correspond to the random selection of coefficients for approximation, and this leads to the conclusion that this is what really makes the configurations measure poorly with the metric. In such a case (coefficients randomly selected), the performance is always bad for any metric, independently of the selected wavelet family. In summary, the reverse biorthogonal wavelet family is the best option for decomposing the images independently of the metric selected for the evaluation; regarding the fusion strategy, there is a correlation between the best option and the selected evaluation metric as indicated above.

**Figure 8:**
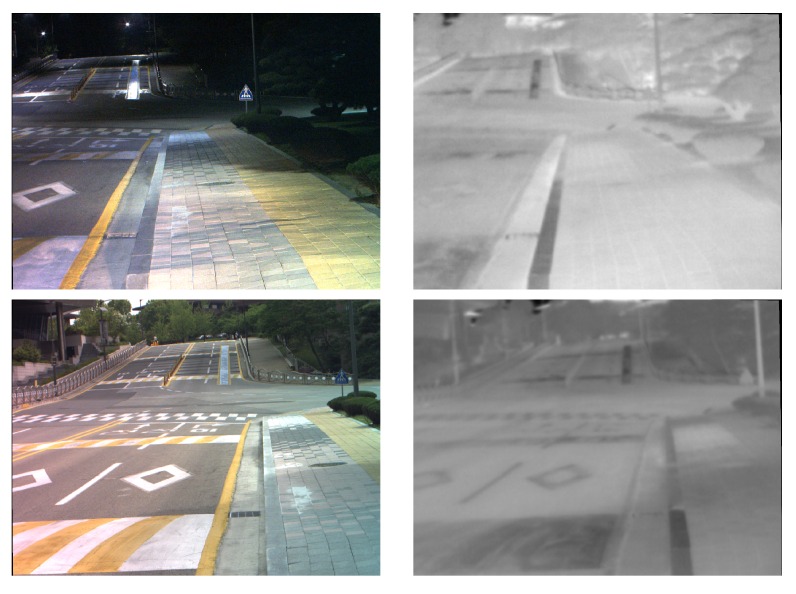

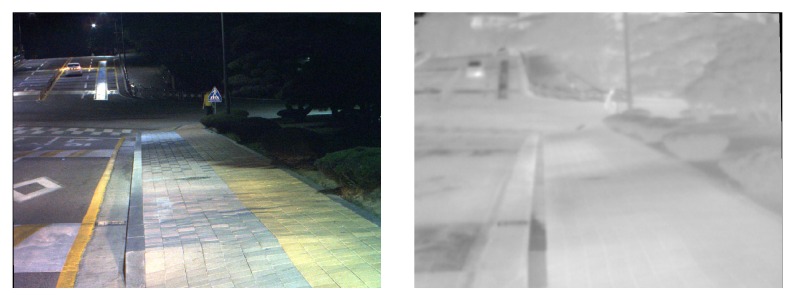
Three pairs of cross-spectral images of the same scene but at different day-time (images from [8]): (**Left**) VS; (**Right**) LWIR.

Finally, a new data set containing visible spectra (VS) and Long Wave Infra-Red (LWIR) images has been considered [8]. This data set contains pair of images from the same scene, taken with the same platform (a color camera from PointGrey and a thermal camera from FLIR), but at a different day-time. Figure 8 presents three pairs of cross-spectral images (VS, LWIR) used to evaluate the needs of having different setups for DWT based image fusion in order to obtain the best performance. These three pairs of images correspond to sequences obtained at: (Top) early morning; (Middle) midday; and (Bottom) late evening. The four evaluation metrics presented in Section 3 have been considered to evaluate the performance of the results obtained from the fusion. The evaluation metrics have a similar behavior than in the previous case; in other words, values from FSS and FS-CIELAB are not that much affected by the different configurations—just the top best setups from the previous test have been considered. On the contrary, the values of FPSNR or FMI metrics have larger variation when they are used to evaluate the performance of the top best setups from the previous test. Having this in mind we can conclude that the values of FPSNR or FMI should be taken into account when looking for the best configurations. Table 4 presents the best setups for the three scenarios presented in Figure 8 according to FPSNR and FMI metrics. These values somehow proof the need of having a different setup according to the characteristics of the images to be fused (in this case day-time) in order to get the best performance. The difference between these best configurations and those obtained with the first data set are mainly due to the nature of the pair of images; the first data set contains VS-NIR pair of images while the second one VS-LWIR pair of images. Such a difference can be easily appreciated comparing the pair of images presented in Figure 7 with the ones presented in Figure 8—LWIR images, also referred in the literature as thermal images, do not have high contrast, they are poor in texture, in comparison with NIR images. It should be mentioned that the objective of the current work is not to find the setup that reaches the best performance, independently of the evaluation metric and kind of cross-spectral image pair, but to identify the most important elements to be considered: (1) one level of decomposition is enough; (2) the approximation weights more than details; (3) from all the wavelet families the rbio is the best choice, its is always among the best configurations (note that in the second data set the rbior family is among the top setups, although the bior5.5 reaches the best performance in the FPSNR evaluation metric).

## 5. Conclusions

This paper presents an empirical study to identify the best setup for discrete wavelet transform (DWT) based image fusion, particularly in the visible and infrared case. In the study a large set of configurations using different wavelet families, decomposition levels and fusion strategies have been compared and quantitatively evaluated. The quality of the fused images has been assessed using several state of the art metrics as well as adaptations proposed in this paper. The obtained results have been validated in a large data set consisting of pairs of registered visible and infrared images, both NIR and LWIR. Future work will be focussed on developing new evaluation metrics which would be computed at a pixel-wise level, combining information from both input images at the same time.

## Figures and Tables

**Table 1:** Setups evaluated in the current work.

Variable	Comments	Values
Wavelet family	Family of wavelet used for both DWT and I-DWT	Haar, Daubechies, Symlets,
		Coiflets, Biorthogonal,
		Reverse Biorthogonal
		Discrete Meyer Aprox.
Level	Level of decomposition	1, 2 and 3
Fusion strategy (approx.)	Strategy used to merge coefficients from both images	mean, max, min, rand
Fusion strategy (details)	Strategy used to merge coefficients from both images	mean, max, min, rand

**Table 2:** Wavelet families evaluated in the current work.

Wavelet Name	Comments	Setups
Haar (haar)	Orthogonal Wavelet with linear phase.	haar
Daubechies (dbN)	Daubechies’ external phase wavelets.	db1, db2, ..., db8.
	N refers to the number of vanishing moments.	
Symlets (symN)	Daubechies’ least asymmetric wavelets.	sym2, sym3, ..., sym8.
	N refers to the number of vanishing moments.	
Coiflets (coifN)	In this family, N is the number of vanishing moments for both the wavelet and scaling function.	coif1, coif2, ..., coif5.
Biorthogonal (biorNr.Nd)	Biorthogonal wavelets with linear phase. Feature pair of scaling functions (with associated wavelet filters), one for decompositions and one for reconstruction, which can have different number of vanishing moments. Nr and Nd represent the number of vanishing moments respectively.	bior1.1, bior1.3, bior1.5,
		bior2.2, bior2.4, bior2.6,
		bior2.8, bior3.1, bior3.3,
		bior3.5, bior3.7, bior3.9,
		bior4.4, bior5.5, bior6.8

Reverse Biorthogonal (rbioNr.Nd)	Reverse of the Biorthogonal wavelet explained above.	rbio1.1, rbio1.3, rbio1.5,
		rbio2.2, rbio2.4, rbio2.6,
		rbio2.8, rbio3.1, rbio3.3,
		rbio3.5, rbio3.7, rbio3.9,
		rbio4.4, rbio5.5, rbio6.8
Discrete Meyer Approximation (dmey)	Approximation of Meyer wavelets leading to FIR filters that can be used in DWT.	dmey

**Table 3:** Performance decrease (percentage) with respect to the best one according to the four evaluation metrics (see Figure 4).

	3% Best FPSNR	3% Best FMI	3% Best FSS	3% Best FS-CIELAB
FPSNR	0.26%	1.17%	**16.79**%	**16.97**%
FMI	2.96%	1.05%	2.24%	3.24%
FSS	**6.39**%	**6.46**%	0.04%	0.17%
FS-CIELAB	2.37%	1.52%	0.008%	0.006%

**Table 4:** Best setups according to the evaluation metric for the pair of cross-spectral images presented in Figure 8.

Day-Time	Evaluation Metric	Wavelet Family	Level	Fusion Strategy (approx. coef.)	Fusion Strategy (details coef.)
Figure 8 (Top)	FPSNR	bior5.5	1	min	max
Figure 8 (Middle)	FPSNR	bior5.5	1	mean	mean
Figure 8 (Bottom)	FPSNR	bior5.5	1	min	max
Figure 8 (Top)	FMI	rbio2.8	1	min	mean
Figure 8 (Middle)	FMI	rbio2.8	1	mean	mean
Figure 8 (Bottom)	FMI	rbio2.8	1	min	max
